# News trends and web search query of HIV/AIDS in Hong Kong

**DOI:** 10.1371/journal.pone.0185004

**Published:** 2017-09-18

**Authors:** Alice P. Y. Chiu, Qianying Lin, Daihai He

**Affiliations:** Department of Applied Mathematics, The Hong Kong Polytechnic University, Hong Kong SAR, China; Brighton & Sussex Medical School, Falmer, UNITED KINGDOM

## Abstract

**Background:**

The HIV epidemic in Hong Kong has worsened in recent years, with major contributions from high-risk subgroup of men who have sex with men (MSM). Internet use is prevalent among the majority of the local population, where they sought health information online. This study examines the impacts of HIV/AIDS and MSM news coverage on web search query in Hong Kong.

**Methods:**

Relevant news coverage about HIV/AIDS and MSM from January 1st, 2004 to December 31st, 2014 was obtained from the WiseNews databse. News trends were created by computing the number of relevant articles by type, topic, place of origin and sub-populations. We then obtained relevant search volumes from Google and analysed causality between news trends and Google Trends using Granger Causality test and orthogonal impulse function.

**Results:**

We found that editorial news has an impact on “HIV” Google searches on HIV, with the search term popularity peaking at an average of two weeks after the news are published. Similarly, editorial news has an impact on the frequency of “AIDS” searches two weeks after. MSM-related news trends have a more fluctuating impact on “MSM” Google searches, although the time lag varies anywhere from one week later to ten weeks later.

**Conclusions:**

This infodemiological study shows that there is a positive impact of news trends on the online search behavior of HIV/AIDS or MSM-related issues for up to ten weeks after. Health promotional professionals could make use of this brief time window to tailor the timing of HIV awareness campaigns and public health interventions to maximise its reach and effectiveness.

## Introduction

The global epidemic of HIV continues to expand. Men who have sex with men (MSM) represents a dominant route of transmission [1, 2]. In Hong Kong, ever since the first case of human immunodeficiency virus(HIV) was reported in 1984 [3], the rate of HIV infections in MSM is rapidly expanding. From 2004 to 2014, the annual number of new HIV cases had increased from 268 to 651, while Acquired Immunodeficiency Syndrome(AIDS) had also increased from 49 cases to 108 cases [3]. Homosexual transmission of HIV was growing fast from 24% to 57% during the same period [3].

Infodemiology is the study of distribution and determinants of information on the internet, and is a powerful tool to inform public health and public policy [4]. Infodemiology methods are either supply-based or demand-based. Supply-based methods analyse online information by classifying them by topic and use indicators to measure changes over time. Demand-based methods use data generated from online search behavior of people such as the number of searches on a specific topic, or the number of visits on websites with a specific topic [5].

Infodemiology had been applied in previous public health studies for various purposes, examples include its use as a syndromic surveillance tool for influenza and dengue fever [6, 7]; as a proxy indicator to measure the awareness of an autism campaign [8]; as an infodemiological tool to study online search patterns for dizziness-related information and prescription drugs [9, 10]; as an infoveillance tool to study the temporal relationships between bariatric surgery-related media events and insurance policy modification [11]; as a tool to measure sudden increases in non-cigarette tobacco product searches following cigarette tax increases [12]; as an impact measurement tool to study the online information seeking behavior following a public figure’s pancreatic cancer announcement [13].

Infodemiological design was also used in HIV/AIDS studies [14, 15]. “HIV” and “AIDS” searches were shown to be correlated with HIV prevalence in different regions of USSR [14]. A recent Canadian study showed that HIV infection rates and HIV-related online campaign activities are positively correlated [15].

The impacts of HIV/AIDS news on awareness, behavior and online searches had been explored. Kalichman et al. reported that following the public announcement of HIV sero-positivity status of basketball star Earvin “Magic” Johnson, there were substantial increases in the awareness of HIV/AIDS, increase in HIV testing and changes in high risk behavior. However, such effects were transient and subsided within three weeks of the announcement [16]. Gabarron et al. demonstrated that public events such as World AIDS Day and actor Charlie Sheen’s announcement of HIV positive status led to increases in Wikipedia searches for HIV-related articles on the day of the event [17].

The relationship between news coverage and online search patterns had also been studied in other diseases. During the Zika virus outbreaks in 2016, information seeking behavior about the disease substantially increased following government announcements [18]. During the weeks following the imported case of Ebola in the United States, there was significant information contagion of Ebola-related media coverage on related tweets and internet searches [19]. Niederdeppe et al’s study found that there was a positive relationship between cancer news coverage and online searches [20].

Clinicians, public health professionals and the general public regularly seek health information on the internet [21]. In Hong Kong, internet uses had reached 73% of the population in 2014. Among them, 96% used the internet for information search [22], and 44% sought health information online [23]. Google had a two-third market share among search engine users in Hong Kong, followed by Yahoo [24]. Google Trends compares the relative search volumes of specified keywords entered by internet uses over time [25]. However, little is known about the impact of routine HIV news coverage on online information searches. Our study aims to explore the temporal trends of news coverage patterns of HIV, AIDS and MSM and examine their magnitude and duration of impacts on relevant online search patterns in Hong Kong from 2004 to 2014.

## Materials and methods

### Google Trends

Google Trends (Google Inc, Mountain View, California, USA) is a publicly available source that provides normalised Google Trends statistics for different queries since 2004. It analyses the Google search queries to determine how many searches were done on the keywords you entered, compared to the total number of Google searches done during that time by all users. Google Trends exclude terms with very low search volumes or duplicate searches made by the same users over a very short period of time. Google Trends are normalised to a scale from 0 (i.e. relative search volume less than 1% of peak volume) to 100 (i.e. relative search volume reaches its peak). We obtained weekly data from January 1st, 2004 to December 31st, 2014, and analysed searches conducted in Hong Kong only. The keywords we considered include “HIV”, “AIDS”, “MSM”, “gay”, “homosexual”, “ai zi bing” (the Chinese term for AIDS), “nan tong xing lian” (the Chinese term for MSM).

### News trends

For news trends, we used WiseNews database as a news extraction tool. WiseNews is a database of newspapers, magazines, journals and newswires published in Hong Kong and other countries. The database covers 18 major local newspapers in both Chinese and English. Our search included four newspapers, Apple Daily, Oriental Daily, Metro Daily and AM730, which represented two paid newspapers and two freely distributed newspapers of top circulations in Hong Kong, from January 1st, 2004 to December 31st, 2014. We used the same set of keywords as with Google Trends. An article is retrieved if it possessed the specified keywords in the news’ headlines or content. Four student helpers assisted in the data extraction process. Each student was responsible for screening half of the time period. Thus, two students worked in parallel and conducted independent screening for each study year. Any discrepancies are resolved by the first author (A.C.).

For an article to be considered relevant, it needs to meet the following inclusion criteria: the news article’s primary topic is clearly related to HIV, AIDS or MSM. For example, it could be a MSM movie review, a scientific discovery related to HIV or AIDS, a sex-related crime committed by a MSM, or a HIV/AIDS awareness event. On the other hand, there is an exclusion criteria that the news article is not related to HIV, AIDS or MSM. For example, it could be news related to Middle East Respiratory syndrome’s drug treatment with a brief mentioning of HIV/AIDS drug treatment as a secondary topic, or news related to MSM committing a crime, but the crime is clearly not HIV/AIDS or MSM-related, such as theft. Each article is scanned for relevance according to the specified criteria by title, and if necessary, the full news article is read and checked.

For each relevant news article, we extracted the following variables into a Microsoft Excel file: type of article (e.g. news, editorial or press release); article topic (e.g. HIV treatment, HIV vaccine, HIV prevention, HIV transmission or risk, HIV surveillance, surveys or statistics, HIV science, HIV publicity events, HIV education or awareness, HIV policy and laws, social issues, sex-related crimes, HIV testing); place of origin (e.g. Hong Kong, Macau, Mainland China, Taiwan or overseas); sub-populations covered (e.g. MSM, lesbians, people living with HIV, general population, celebrities); and total number of news articles (i.e. the number of articles published on the same issue or topic on the same date).

### Pilot study and inter-rater reliability

We conducted a pilot study where four student helpers independently extracted news articles using the methods described above, in the month of December 2004. We computed Kappa coefficient for inter-rater reliability (IRR) for each variable collected [26].

### Data compilation and statistical analyses

Following the data extraction process, we created Google Trends time series and WiseNews Trends that represent aggregate time series and categories that are classified as news article type, news topic, news origin or population groups from 2004 to 2014. Data are then imported to R Statistical Software version 3.4.0 (https://cran.r-project.org/) for statistical analyses.

The steps for analysing the causality between news trends and Google Trends are as follows. We first applied moving average models to decompose Google Trends into three time series- trends, seasonal and random factors. Anderson Darling tests were then used to test for normality of the decomposed random factors. Augmented Dickey Fuller tests were used to test for stationarity of the decomposed random factors. The number of lags was determined based on four information criteria: Akaike Information Criterion (AIC), Schwarz Criterion (SC), Hannan-Quinn (HQ) and Final Prediction Error (FPE). We selected the lag which is the mode determined by these four criteria. Based on the time lag, the cause-effect relationships of news trends on Google Trends can be analysed by applying the Granger Causality tests. This statistical test does not rely on a model specification and therefore is suitable for empirical investigations of cause-effect relationships [27–29]. Granger causality test is suitable for analysing our data because of the following assumptions: (i) The effect (i.e. Google Trends) does not precede its cause temporally. (ii) The causal time series (i.e. Google Trends) contains unique information about the time series being caused (i.e. news trends that is not available otherwise) [27–29]. Statistically significant pairs of time series were further explored using impulse response function. Essentially, we investigated the reaction in Google Trends in response to changes in the news trends. Statistical significance was assessed at the 5% levels.

This study involves secondary data collection and data analyses only, and human participants are not involved. Ethical approval was not needed.

## Results

### Inter-rater reliability

We determined IRR during the pilot study. Four student helpers independently extracted, screened and recorded WiseNews articles from December 1st, 2004 to December 31st, 2004. 160 news records were retrieved. Kappa coefficients for different variables were computed as follows: publication date(1.00); number of articles(0.608); news origin(0.375); population group studied(0.530); article type(0.430); and article topic(0.192).

### Impact of news trends on Google Trends

**Fig 1:**
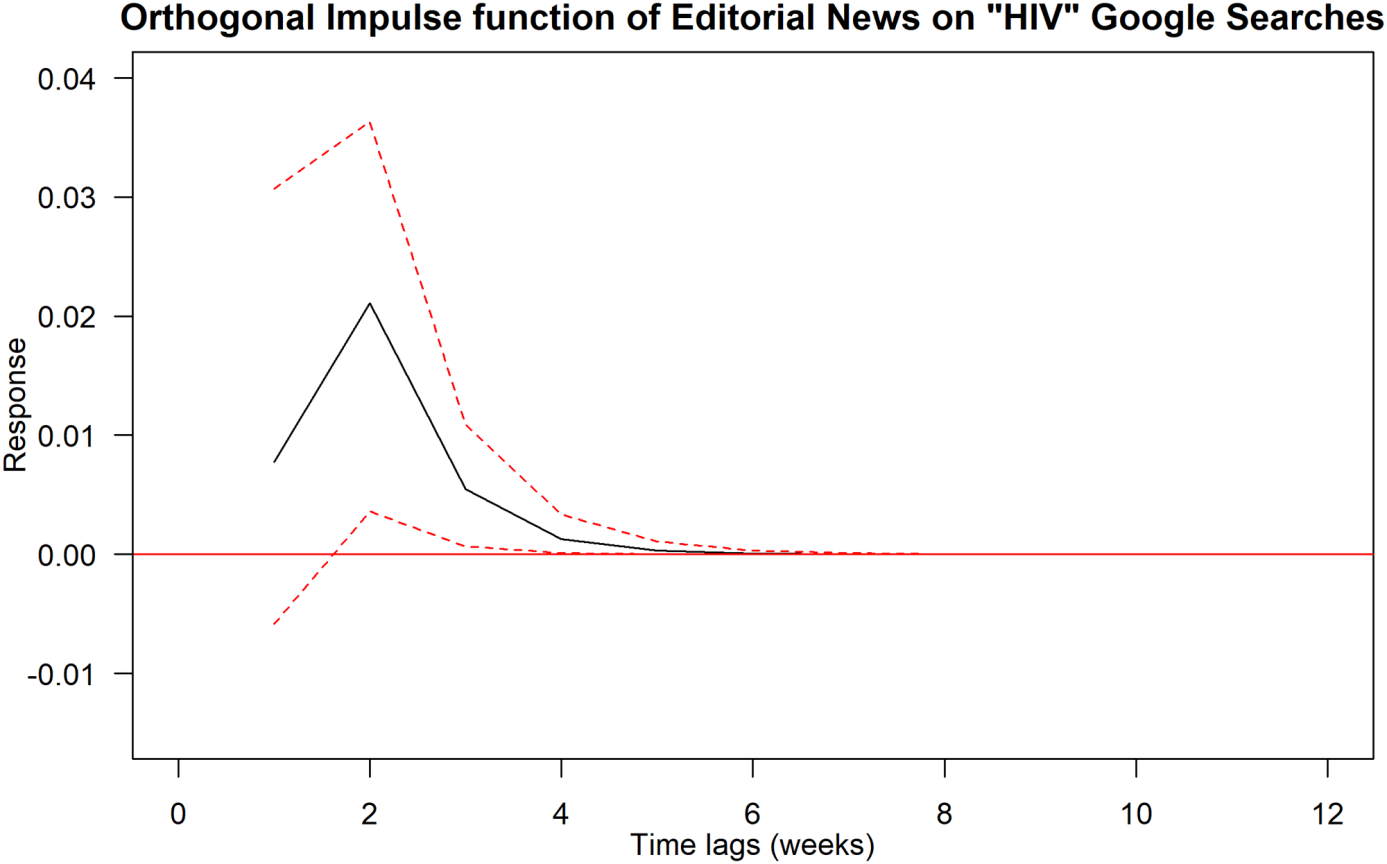
Orthogonal impulse function of editorial news trends on HIV Google Trends. Black solid line shows the response curve. Red-dashed line indicates the 95% confidence interval.

**Fig 2:**
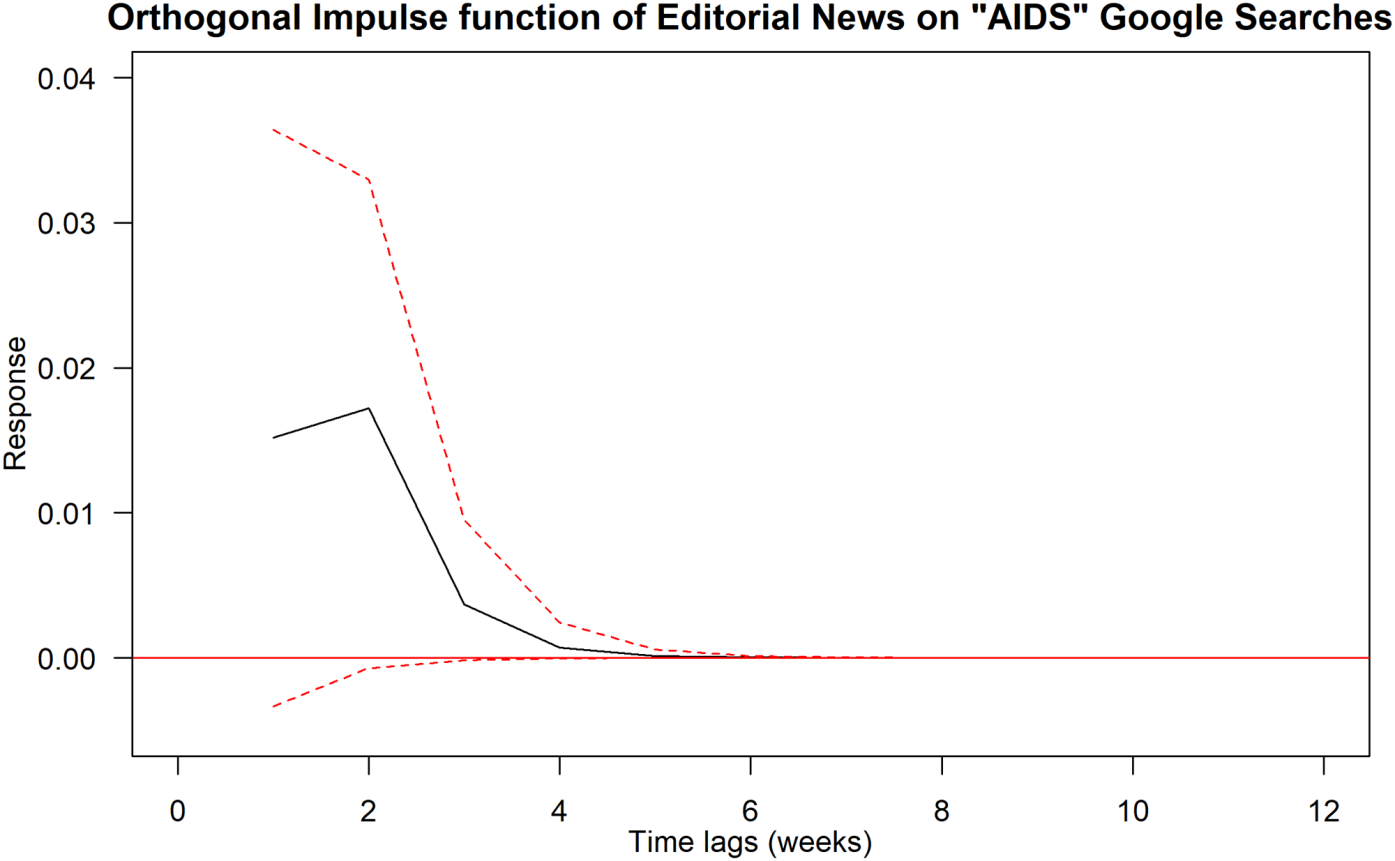
Orthogonal impulse function of Editorial news on AIDS Google Trends. Black solid line shows the response curve. Red-dashed line indicates the 95% confidence interval.

**Fig 3:**
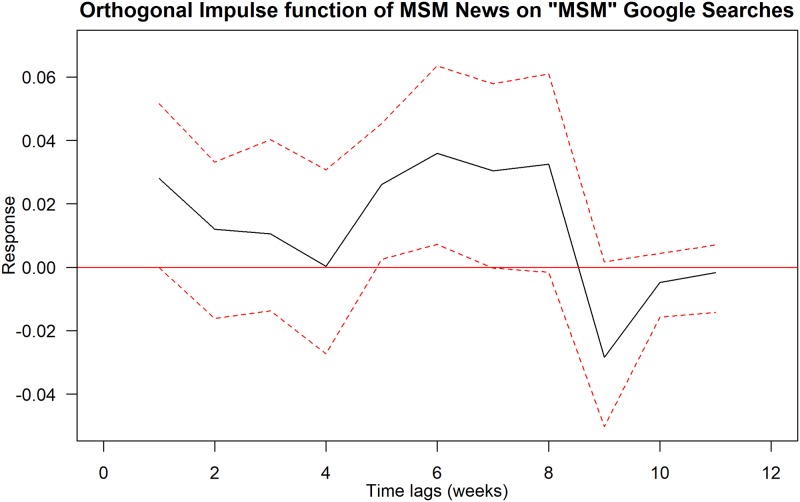
Orthogonal impulse function of MSM-related news trends on MSM Google Trends. Black solid line shows the response curve. Red-dashed line indicates the 95% confidence interval.

Using Granger Causality tests, we found that the following pairs of news trends and Google Trends are statistically significant at 5% levels: (i) editorial news trends on HIV Google Trends; (ii) editorial or other news trends on AIDS Google Trends; and (iii) MSM-related news trends on MSM Google Trends. We further explored these relationships using impulse response function and the results are summarised in Figs 1–3.

Fig 1 shows the orthogonal impulse function of Editorial news trends on HIV Google Trends. Note that the impact of editorial news on “HIV” google searches increases rapidly and reaches its peak on average of two weeks after the news are published. The impact decreases rapidly three weeks after and declines gradually to zero level six weeks after.

Fig 2 displays the orthogonal impulse function of Editorial or other news on AIDS Google Trends. Again, we found that the impact of Google Trends increases after one week and reaches its peak after two weeks. It declines abruptly three weeks after. It reaches zero level five weeks after.

In Fig 3, we show the orthogonal impulse function of MSM-related news trends on MSM Google Trends. We found that there is a more fluctuating impact after one week. It drops to zero level four weeks after, but increases again and reaches its peak impact six weeks after. It continues to fluctuate around the peak level seven and eight weeks after. The impact suddenly drops to negative nine weeks after. The impact is slightly negative ten weeks after but returns to zero level 11 weeks after.

## Discussion

To the best of our knowledge, this study is the first to examine the relationship between news trends and search engine queries on the topics of HIV, AIDS and MSM. We found that editorial news have a positive impact on searches on both HIV and AIDS. Also, news trends on a MSM-related topic had a predominantly positive impact on search behavior on the same topic. Such impacts lasted for up to ten weeks. Such observation is supported by the channel complementarity theory that was proposed by Dutta-Bergman [30, 31]. This theory implies there is a congruence between the online search behavior and news media within a specific topic [30, 31]. We could also apply this theory in our context to explain why HIV news coverage could lead to online information searches.

Gabarron et al. found that the number of HIV queries on Wikipedia increased significantly during international public events such as World AIDS Day and the announcement of the HIV-seropositivity of actor Charlie Sheen [17]. Consistent with their findings, our study further showed that Google online searches has a lagged time impact of up to ten weeks after the publication of HIV-related news.

The temporal relationships between health news and online searches had also been demonstrated in cancers [20], Ebola [19] and mammography screening [32]. Niederdeppe et al. noted a positive relationship between cancer news coverage and information seeking [20]. Towers et al. found evidence of contagion of fear during the Ebola outbreaks, where Ebola-related news videos would lead to tens of thousands of Ebola-related internet searches and tweets [19]. Weeks et al. suggests that news coverage of a mammography guideline may have driven online information seeking. In line with previous research, our study demonstrates that news coverage of HIV, AIDS or MSM related topic will stimulate online searches on these topics.

Our study has important public health implications. Health authorities and AIDS-related non-governmental organisations can make use of the time window of increased online search interest to promote awareness and provide public education on this topic [18]. Awareness campaigns, publicity events, preventive education and other health promotion activities could be implemented after significant HIV, AIDS or MSM news to attract more public attention.

Our study has several key strengths. First, our findings are novel in identifying the duration of time-window for online search increases following HIV news coverage. Second, we used triangulation methods which employed both qualitative and quantitative techniques. Our news searches are screened for content relevance by student helpers. In contrast, previous studies mostly used keyword matching techniques without considering the content relevance [20]. Third, our study could be conducted at a low cost and cover a wide study population.

Our study is limited by several factors. First, only aggregate data were available. We could not confirm whether news and media coverage could indeed impact online searching behavior at the individual level. Second, we have included only four newspapers rather than a comprehensive coverage of all local newspapers in Hong Kong, although we believe our selection of two free and two paid newspapers of top circulation should cover a broad readership. Third, the IRR of the news coverage searches is only satisfactory.

## Conclusions

This infodemiological study shows that news trends have a positive impact on online search behavior on HIV, AIDS or MSM-related issues. Health promotional professionals could make use of these insights to design timely online awareness campaigns and public health interventions that will maximise reach and effectiveness. Our research techniques could be applied to study other sexually transmitted diseases. Our results have implications on the timing of disseminating HIV, AIDS and MSM health information online.

## Supporting information

S1 Table HIV Google Trends. (XLSX)

S2 Table AIDS Google Trends. (XLSX)

S3 Table MSM Google Trends. (XLSX)

S4 Table WiseNews coverage 2004–2014. (XLSX)
